# Implications of introducing case based radiological images in anatomy on teaching, learning and assessment of medical students: a mixed-methods study

**DOI:** 10.1186/s12909-022-03784-y

**Published:** 2022-10-14

**Authors:** Ramya Rathan, Hossam Hamdy, Salah Eldin Kassab, Miral Nagy F. Salama, Anusha Sreejith, Aji Gopakumar

**Affiliations:** 1 College of Medicine, Gulf Medical University, Ajman, UAE; 2 Department of Physiology, Faculty of Medicine, Suez Canal University, Ismailia, Egypt; 3 Data and Statistics Department, Emirates Health Services, Dubai, UAE

**Keywords:** Anatomy, Radiological images, Item analysis, Student perception

## Abstract

**Background:**

Introducing radiological anatomy in the preclinical curriculum can increase the understanding of Anatomy**. **Regardless of the integration when teaching anatomy, it is essential to maintain oversight as to what and how much is being taught. In addition, the knowledge requirements for preclinical students should be considered. The purpose of this kind of integration is that the student should be able to apply the knowledge which can help them better understand anatomy and not to make the course more challenging. This study aimed to understand whether adding radiological images would increase the difficulty level of the questions.

**Methods:**

We introduced radiological images, including X Rays, CT scans and MRIs, when teaching anatomy in the preclinical curriculum. A class of 99 students were tested using A-type MCQs (*n* = 84). All 84 questions were categorized on whether they were case-based with or without a radiological image. The item analysis of both groups of test questions was then compared based on their difficulty and discrimination index. A qualitative student perception regarding the inclusion of radiological images in anatomy was also measured using a questionnaire with a 5-point Likert scale.

**Results:**

The results showed that the performance level of the students was similar when comparing the test questions in both groups. The item analysis of the MCQs in the two groups revealed that by integrating radiological images when teaching anatomy, the various parameters in both groups of test questions were in the same range. More than 80% of the students felt that radiological images facilitate the achievement of learning outcomes and help to apply their knowledge in clinical contexts. The study's findings reported that the rate of satisfaction by including radiological images when teaching anatomy is high.

**Conclusion:**

Recognition and interpretation of images are essential in an undergraduate medical program. Students found it helpful when radiological images were introduced to them when teaching anatomy. Since the students' performance in summative exams in both groups of questions was in the same range, the findings also point out that adding radiological images when teaching anatomy does not increase the difficulty of the subject.

## Background

Anatomy, the basis of modern medicine [1], is considered one of the cornerstones and the foundation of clinical skills in the medical curriculum [2]. Anatomy requires learning approaches beyond the rote learning of structures, which is achieved by innovation in teaching methods. Although there is no doubt about the importance of teaching, the argument on how best an instructor could provide this teaching continues. This argument has been increased by the shift in the curriculum worldwide to competency-based education by changing the curriculum from knowledge acquisition to knowledge application [3, 4]. Furthermore, this led to many medical schools putting much effort into integrating clinical experiences at the beginning of medical school [5]. One of the ways this was carried out was by incorporating radiological images in course materials [6, 7]. Incorporating these images further needs to be measured to ensure that the intended learning outcomes are met. Most medicine disciplines use case-based items for student assessment [8]. A case-based item is said to be theoretically broader in scope, require assimilation of more content, and can be categorized at the higher cognitive levels in Bloom's taxonomy [8]. Therefore, it is essential to evaluate the items to determine the good from the bad. This can be measured using two parameters from the item analysis report: the difficulty index and the discrimination index. These two parameters are used to qualify and determine the inclusion of an item in various standard licensing examinations.

A literature search concluded that there was minimal emphasis given to the role of images in Assessments [9–11]. Also, there was not much importance given to the difficulty index and the discrimination index of including radiological images on the student's performance. A study conducted in 2014 suggested that the process in which the questions that contained images were answered required different cognitive processes [12]. Sweller (1994) said that the interpretation of an image could add to the student's cognitive load [13]. If the student has no appropriate schemas, that can add to the extraneous cognitive load to interpret the Image in the question.

Regardless of integrating radiology when teaching anatomy, it is vital to maintain oversight regarding the content taught. In addition, one should consider the knowledge requirements needed for preclinical students. The purpose of this kind of integration is that the student should be able to apply the knowledge which can help them better understand anatomy and not to make the course more challenging. The study aims to measure the effect of teaching radiological anatomy on medical students' learning and performance on high stake exams. The research questions posed were what is the effect of introducing radiological anatomy teaching on the student performance in summative anatomy examinations? Would the addition of radiological images increase the difficulty level of the questions on the students? What were medical students' perceptions about introducing the teaching of radiological anatomy?

## Methods

This study used a mixed-methods design approach. For the present study, 99 students of the 2018 class enrolled in the MBBS program at Gulf Medical University were selected. Phase II of the MBBS program spans the 2nd and 3rd year MBBS and includes the pre-clerkship phase with modular organ-system modules. Concepts of Basic Medical Sciences (Anatomy, Physiology, Biochemistry, Pathology, Microbiology, Pharmacology, Community Medicine and Forensic medicine) are integrated throughout the phase.

Since the study population targeted the preclinical science years, a guiding principle was set in developing the course content and the teaching and assessment strategies. The common problems related to the system were identified. The normal anatomy consists of structures that must be identified on common modalities and cross-modality correlations' importance. Once this was completed, the common pathologic conditions and findings that students should see were also explained to the students. The detailed topics that were included in the study plan are shown in Table 1.

**Table 1:** Radiological topics included in the study plan in different modules

Region	Objectives Specified	Radiological Topics	
		**Normal**	**Pathological**
**Musculoskeletal Module**	• Label the bones of both limbs and identify their parts seen in the **X- ray** • Identify the joints of the limbs seen in **X-** ray and CT scan • Recognize the difference between the shoulder and hip joints in normal CT scan • Differentiate between the anterior and posterior view of the normal knee with emphasis of cruciate ligaments seen in MRI • Count the vertebrae both posteroanterior and lateral view seen in (X-ray, CT scan and MRI) • Recognize the common bone fractures of both limbs (X-ray) • Identify the joint dislocations of both limbs and their direction seen in (X-ray, CT scan) • Identify the injured cruciate ligament in MRI of the knee • Identify the abnormal spine curvatures like kyphosis and scoliosis seen in (X-ray, CT scan) • Differentiate between spondylolisthesis and vertebral dislocation seen in CT scan and MRI spine • Recognize the herniated disc and determine its level in sagittal sections of CT scan and MRI	• Introduction to Radiology and (orientation of different X-ray views, different CT scan and MRI sections) • Identification of bones of both extremities and their different parts in X- rays • Identification of normal joint appearance of both extremities in X-ray • Normal appearance of the spine in the cervical, thoracic, lumbar, and sacral regions seen in X-ray, CT scan and MRI	• Fracture of clavicle, mid shaft and supracondylar part of humerus, radius, and ulna seen in X-ray • Fracture of hip, acetabulum, neck of femur, tibia, and fibula seen in X-ray • Dislocation of shoulder and elbow seen in X-ray and CT scan • Dislocation of hip seen in CT scan • Tear in cruciate ligaments of the knee seen in MRI • Abnormal curvature of the spine seen in X-ray and CT scan and MRI (Frontal & sagittal section) • Vertebral dislocation, spondylolisthesis of the spine and disc herniation seen in sagittal CT scan and MRI
**GIT Module**	• Identify the lumbar vertebrae, sacroiliac joint and Psoas shadow seen in X-ray • Identify the kidney shadows, intestinal gases seen in X-ray • Identify the normal appearance of the alimentary canal using different barium studies • Recognize the different abdominal organs in relation to each other in axial CT scan sections with and without contrast • Identify some of the congenital anomalies of the digestive system through X-ray like anal atresia and ultrasound like congenital duodenal atresia • Recognize the appearance of esophageal, gastric, and intestinal abnormalities e.g., esophageal atresia, hypertrophic pyloric stenosis, peptic ulcer, Hirschsprung disease seen in barium studies • Identify the appearance of tumors of the digestive system seen in CT scan with and without contrast	• Plain X-ray of abdomen (Identify lumbar vertebrae, sacroiliac joint, Psoas shadow) • Introduction to barium studies and procedure (Barium swallow, barium meal, barium meal follows through, barium enema) • CT scan of the abdomen at different levels with and without contrast	• Xray for anal atresia and ultrasound for duodenal atresia • Barium studies for esophageal atresia with fistula, achalasia, gastric and duodenal ulcers, Chron's disease, intestinal obstruction, volvulus and Hirschsprung disease • CT scan for liver, pancreatic carcinoma
**Urinary System and** **Reproductive system**	• Identify important pelvic landmarks like sacro -iliac joint and ischial spine • Identify the normal appearance of urinary system and female reproductive system seen in intravenous pyelography and hysterosalpingography respectively • Identify the normal appearance of renal artery in CT scan angiography • Identify the normal appearance of testis, epididymis, their vasculature, and the uterine parts seen in ultrasound • Identify the normal appearance of kidney in abdominal CT scan and uterus in pelvic CT scan • Renal, ureteric stones, hydronephrosis seen in X-ray and IVP • Identify the narrowed renal artery seen in CT scan angiography • Identify the appearance of renal tumors, polycystic kidney, hydronephrosis, renal ptosis in CT and MRI • Identify the uterine fibroids & polycystic ovary in ultrasound and uterine carcinoma in CT scan • Identify the blockage of uterine tube and some congenital anomalies of the uterus like bicornuate, unicornuate and septate uterus and blocked uterine tubes seen in hysterosalpingography • Identify the Torsion of the testis, varicocele, hydrocoele and testicular tumors seen in ultrasound	• Normal appearance of pelvic landmarks seen in X-ray • Normal appearance of kidneys, ureters and urinary bladder seen in IVP & normal appearance of tubes, uterus and cervix in hysterosalpingography • Normal appearance of renal blood vessels in CT scan angiography • Normal appearance of testis, epididymis, their vasculature, and the uterine parts seen in ultrasound	• Renal, ureteric stones, hydronephrosis seen in X-ray and IVP • Renal artery stenosis seen in CT scan angiography • CT and MRI in cases of (Tumors, Polycystic kidney, Hydronephrosis, Renal Ptosis, Reverse Rotation of the kidney) • Uterine fibroids & polycystic ovary in ultrasound and uterine carcinoma in CT scan • Some congenital anomalies of the uterus like bicornuate, unicornuate and septate uterus and blocked uterine tubes seen in hysterosalpingography • Torsion of the testis, varicocele, hydrocoele and testicular tumors seen in ultrasound
**Respiratory system** **Cardiovascular system**	• Identify the nose, paranasal sinuses in skull X-ray, CT scan • Differentiate between the posteroanterior and anteroposterior views of chest X-ray • Identify cardiac, mediastinal, and respiratory shadows in the different X ray views of the chest • Identify the cardiac borders, apex of the heart, aortic knuckle, cost diaphragmatic angles, trachea, and lung shadows in the posteroanterior view of X-ray • Able to mark the different lung lobs in lateral and posteroanterior views of X-ray • Identify the normal appearance of cardiac chambers and their relation to each other in, the big vessels and lungs axial CT scan and echocardiography • Recognize the appearance of sinusitis seen in X-ray and frontal CT scan • Recognize the shifted mediastinum, pericardial, pleural effusion, cardiomegaly, foreign bodies in the lungs, lung atelectasis, lung abscesses and lung tumors seen in Xray and CT scan • Identify some of the congenital anomalies of the heart like egg on string in transposition of great vessels, Coeur en sabot in Fallot's tetralogy and rib notching in coarctation of aorta seen in X-ray • Identify ASD, VSD and PDA seen in echocardiography	• Normal appearance of nose and paranasal sinuses seen in the Xray and CT scan • Normal appearance of cardiorespiratory organs seen in chest Xray, CT scan with and without contrast	• Appearance of cardiomegaly pericardial, pleural effusion, pneumothorax, lung atelectasis, foreign body, lung abscesses and tumors in chest X-ray, CT scan with and without contrast • Appearance of some congenital cardiac malformation in chest x-ray like Fallot's tetralogy, transposition of great vessels and coarctation of aorta seen in X-ray, other anomalies like ASD, VSD and PDA seen through echocardiography
**Nervous System**	• Identify the various lobes and ventricles of brain seen in sagittal, axial, and frontal views of CT scan and MRI • Identify the parts and major landmarks of the brain stem and cerebellum in Sagittal view of CT scan and MRI • Differentiate between the extradural and subdural hemorrhage in axial CT scan and compare both to the intracerebral hemorrhage • Recognize the ventricular dilatation of ventricles in CT scan and MRI and differentiate between the communication and non-communicating types of hydrocephalus • Recognizing abnormal intracerebral masses whether benign or malignant and compare them to the infectious mass like brain abscesses seen in CT scan and MRI	• Normal appearance of brain, brain stem, cerebellum and ventricular system in various CT and MRI sections	• Extradural, subdural, and intracerebral hemorrhage seen in CT scan and MRI • Brain abscess, intracerebral masses whether benign or malignant seen in CT scan and MRI
**Endocrine system and breast**	• Identify the normal appearance of and parts of Sella turcica in lateral X-ray and normal appearance of pituitary gland in relation to the surrounding structures in sagittal & frontal sections of CT scan and MRI • Identify the normal appearance and parts of thyroid gland in ultrasound • Identify the normal appearance of the breast in ultrasound and mammography • Identify double floor Sella turcica seen in X-ray • Recognize pituitary tumors seen in CT scan and MRI • Identify thyroid nodules seen in ultrasound and thyroid tumors seen in CT scan • Identify adrenal gland tumors like pheochromocytoma seen in frontal and axial CT scan sections • Identify breast masses seen in mammography and ultrasound	• Parts of Sella turcica in lateral X-ray and normal appearance of pituitary gland in sagittal & frontal sections of CT scan and MRI • Normal appearance of thyroid gland in ultrasound • Normal appearance of the breast tissue in ultrasound and mammography	a. The double floor Sella turcica (due to pituitary tumor) seen in X-ray b. Pituitary tumors and their pressure effects on the related structures like optic chiasma and sphenoid sinus seen in CT scan and MRI c. Thyroid nodules seen in ultrasound and thyroid tumors seen in CT scan d. Adrenal gland tumors like pheochromocytoma seen in frontal and axial CT scan sections e. Breast masses seen in mammography and ultrasound

**Table 2:** Examples of scenario based MCQs, with and without radiological Images

Questions	Likert scale (1—Strongly disagree; 5—Strongly agree)				
	**1**	**2**	**3**	**4**	**5**
**Meeting radiological learning outcomes**					
RA learning was useful in helping me to identify and describe normal radiological features					
RA learning was useful in helping me differentiate normal from abnormal radiological anatomy					
**Application and contextualization of anatomical knowledge**					
RA learning helped me apply my knowledge of anatomy in a clinical context					
RA learning helped me identify areas of anatomy where my knowledge and understanding are insufficient					
RA learning helped me revise anatomy					
**Familiarity and interest in RA**					
RA learning helped me become familiar with radiological imaging					
RA learning has increased my interest in learning anatomy					
**Free Comments:**					

^*^RA Radiological anatomy

During the second half of each module, sessions on radiological anatomy were delivered to the students. By this time, the students would have acquired an orientation to viscera and bones from the dissection and theory classes. The radiological sessions comprised variable radiological imaging modalities, which included X-rays, CT scans, ultrasound images, and MRIs shown in Table 1. The session was conducted using powerpoint presentations. The content of each session was based on the must‑, should‑, and desirable‑to‑know clinical conditions related to the prescribed university syllabus.

**Fig. 1:**
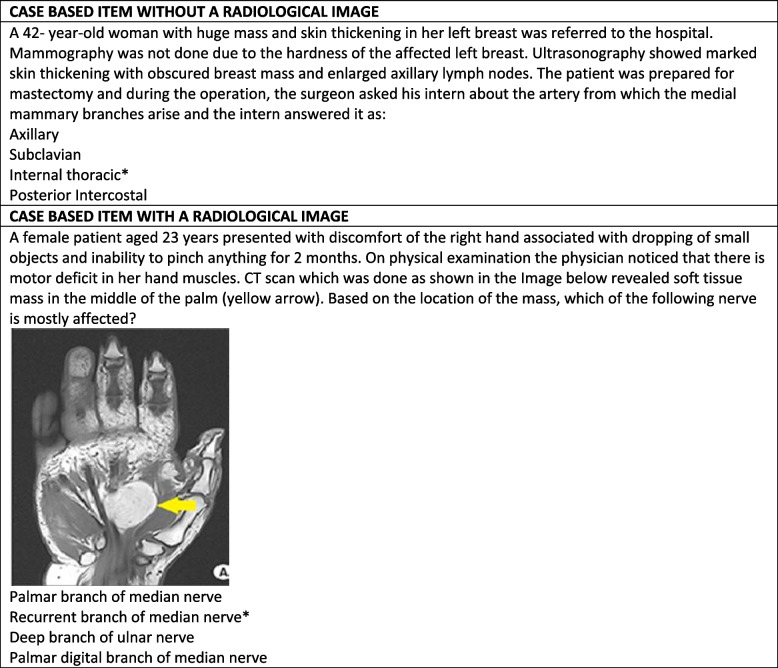
Examples of scenario based MCQs, with and without radiological Images

The evaluation was done at two levels:

- *Comparing the students' performance through MCQs*: At the end of each module, an end-module examination was conducted using MCQs. All the test items of Anatomy were written by the subject expert and were further validated by peer review. All items were written in a single best answer format (A-type) with a rich stem lead-in statement and four response options. Multiple choice questions from seven end module exams from the fall semester of 2019 to the spring semester of 2021 provided the basis of this study. From each end module exam, the questions related to anatomy were extracted. A total number of 84 questions were obtained. Each test item was then reviewed and categorized as either image-based or without a radiological image. For image-based items where scenario-based format with a radiological image, the student had to analyze the scenario correctly, refer to the attached Image and make an informed choice using a reasoning process. On the contrary, an item without a radiological image where a case-based scenario which could be answered. An example of the same is shown in Fig. 1.
- *Student Perception (Kirkpatrick level 1)*: The student's perception was collected using a self-administered questionnaire. The students were asked to answer the questionnaire after the assessment of all the modules. The questionnaire, as shown in Table 2, consisted of 8 items and students were asked to rate the teaching and learning program. For all items, a five-point Likert scale (1, strongly disagree; 5, strongly agree) was used to express the students' opinions. The items in the questionnaire were categorized into three themes which were a) Facilitating the achieving of learning outcomes, b) Application and contextualization of anatomical knowledge and c) Familiarity and interest in radiological anatomy. Also, free responses were used to assess students' opinions further. Finally, feedback was evaluated by summating the scores for each item.

The questionnaire was evaluated in three steps: a) the study questionnaire was adapted from a previously published article [14]. b) The questionnaire was modified for the current study context. Finally, a group of faculty members (*n*=5) evaluated the questionnaire and provided feedback on the clarity and relevance of the questionnaire items in measuring the aimed construct. c) The final version was piloted with 12 students.

### Data analysis

Descriptive statistics and inferential techniques were applied to achieve the study objectives. The descriptive statistics included a measure of averages(mean), a measure of dispersion (standard deviation), a measure of correlation (Point biserial) and the percentages of items with difficulty levels. The Kolmogorov–Smirnov test investigated the normality of the data. After testing for normal distribution, an independent sample t-test [2-tailed] was used to calculate the mean test scores and standard deviations (SD) in the two groups of questions, i.e., the group with and without radiological images. For all statistical tests in this study, a *p*-value smaller than 0.05 was set as the minimum criterion for statistical significance. The analysis was conducted using the statistical package for social sciences (SPSS), version 20.0 (IBM Corp., Chicago, IL).

Test item analysis indices included item difficulty, item discrimination and the point biserial correlation were calculated for each of the 84 questions in each group. The difficulty index was calculated as the percentage of correct responses. It can be calculated by the formula: (H + L / N) × 100, where H represents the number of students who correctly answered the question in the high achieving group. L represents the number of students who correctly answered the question in the low achieving group, and N is the total number of students [15]. The Discrimination index refers to the difference between the average grade of the students with the highest totals to the item total grade and the average item grade of the students with the lowest totals relative to the item total grade divided by the number of students in a quartile The formula can calculate it: (H—L / N) × 100. Point biserial measures the item discriminative power; this indicator compares performance on an item relative to the whole test performance [16].

Piloting the questionnaire discovered that the students clearly understood the words used in the questions. It also determined that an average time between five and ten minutes was required to complete the questionnaire. The reliability of the study questionnaire which was measured using Cronbach's Alpha was found to be 0.92. The percentage of students' agreement was determined and was calculated by summing up the percentage of students who strongly agreed and agreed with the given items. The results were presented in tables and graphs. For the qualitative data, the free responses from the open-ended questions were collected and grouped based on the three themes of the questionnaire. Any overlapping response was deleted, and the remaining data were used to interpret the free responses.

## Results

### Test item analysis

Forty two percent of the questions consisted of a radiological image. Kolmogorov–Smirnov test tested all the item analysis results for normality and found that the variables follow a normal distribution. Table 3 shows the mean and standard deviations for these parameters and compares the means between the two groups using the independent samples t-test [2-tailed]. The values in Table 3 showed that the *p*-value of the difference in difficulty level between the two groups was 0.87, which implies that the difficulty of the test items was not affected by adding a radiological image to an MCQ item. Similar results were seen for the discrimination index and point biserial. Mean values showed that the discrimination index for items appeared unaffected by adding an image within the stem (0.43 ± 0.19 vs 0.36 ± 0.19; *p* = 0.09). Item point biserial correlation, which is a measure of item discrimination and is the correlation between the item score and the total test score, also showed no difference in means between these two groups (0.36 ± 0.15 vs 0.32 ± 0.15, *p* = 0.15).

**Table 3:** Comparison of average test item analysis indicators among the two groups

	**Mean ± SD**		
	No Radiology	Radiology	*P* value
Difficulty Index	0.50 ± 0.20	0.49 ± 0.20	0.873
Discrimination Index	0.43 ± 0.19	0.36 ± 0.19	0.10
Point Biserial	0.36 ± 0.15	0.32 ± 0.15	0.158

### Analysis of students' perceptions

Overall, the Likert scale and the free responses indicated that students felt that incorporating radiological images when teaching anatomy was effective and allowed them to understand anatomy better. Out of the 99 students enrolled in the course, 72 completed the questionnaire and 59 students commented in the free response section.

### Facilitate achieving of learning outcomes

Overall, 32% of the respondents" strongly agreed", and an additional respondent, 59% ", agreed" that the radiological sessions helped them to identify and describe typical anatomical features. Most of the students (60%) "agreed", and an additional 29% "strongly agreed" that these sessions were helpful and helped them to differentiate normal from abnormal features on the image compared to 4% of students who "disagreed". The free responses revealed that students felt radiological Images were essential in learning the organ system and helped them apply their knowledge clinically. Nevertheless, they would appreciate more sessions and practice learning to read radiological Images.

### Application and contextualization of anatomical knowledge

Overall, 50% of the respondents "agreed," and an additional 38% "strongly agreed" that the radiological sessions helped them apply their knowledge of Anatomy in clinical contexts. Most students were also satisfied with the number of sessions conducted in each module. 54% of the students "agreed," with an additional 21% who "strongly agreed" that the number of questions with radiological images in the end module exams was adequate. Some of the free responses mentioned were that students felt only specific pathologies were focused on.

### Familiarity and interest in RA

Overall, 47% of the respondents "agreed," and 38% "strongly agreed" that learning radiological anatomy helped the students become familiar with different radiological imaging modalities. In addition, 46% of the respondents "agreed" that learning radiological anatomy helped the students increase their interest in learning anatomy. Finally, 38% of the students "agreed," and 25% "strongly agreed" that the inclusion of radiological anatomy helped them perform better in the end module exams and the IFOM progress test. Free responses revealed that implementing radiological images had instilled an interest in radiology.

## Discussion

Changes in the curriculum are slow, and much evidence-based practice is emerging. There are several previous studies which demonstrated the importance of integrating radiology into the undergraduate curriculum [17–19]. Since radiological modalities such as CT scans and Ultrasound images are becoming more common in various diagnostic and invasive procedures, medical education emphasizes more on the incorporation of such topics, which are related to radiological images [20–23]. A study in 2015 concluded that when radiology was taught in the preclinical years, students gained a higher understanding of the subject [24]. Accreditation bodies and academic and educational administration also focus on early clinical exposure. The "foundation curriculum" of the academy of the royal colleges also emphasizes the core skills required for the foundational trainees, which says that a foundation doctor should be able to confirm the clinical findings early by asking and interpreting the results of appropriate investigations [25]. A study in 2003 suggested that, most frequently, a physician encounters the internal structure of the human body through radiological images [26]. This shows that many fundamental principles behind medical reasoning can be understood through radiological images.

Practical utilization of the knowledge of anatomy usually happens in the later years of medicine, in which the practical aspects of radiological images are taught [27]. This, in turn, has resulted in a vast knowledge gap between knowledge acquisition in anatomy and the chances of using it in practice. We believe this study will provide an additional resource and a basic guide to integrating radiology when teaching gross anatomy to students in the preclinical years of medicine. It can increase the student's awareness and exposure and benefit their long-term clinical training. As the population in this study were students from the preclinical years of the MBBS program, they gained exposure to different radiological modalities. One of the comments in the qualitative analysis given by a student also mentioned that this kind of intervention developed an interest in choosing radiology as a career option. An earlier study mentioned that those medical students exposed to radiology in the preclinical years are less likely to believe negative stereotypes about radiologists due to a greater awareness of radiology [24]. Also, by having a better understanding of radiology, a non-radiologist physician can improve patient care by promoting positive interactions and using appropriate diagnostic tests [28]. Introducing radiological images to students can help them understand how each organ appears on those images. Exposing the students to this kind of Image in the early years of medicine will help them acquire the essential skills to interpret an image so that they are prepared for their post-graduate training and will help them deal with more challenging topics in the future.

Due to the increasing interest in including images when teaching anatomy [29], the present study aimed to measure the effect of including radiological images. Many studies used students' perceptions regarding the use of radiological images in anatomy and concluded that it enhanced the quality and efficiency of anatomy instruction [30–33]. According to the results obtained in our study, it was evident that the rate of satisfaction of the students by including radiological images when teaching anatomy is positive, which was similar to the results obtained in previous studies [34–37].

Students considered the use of including radiological images when teaching anatomy to be highly effective and of essential importance towards gaining knowledge in anatomy.

Radiological images form a crucial element in anatomy; the students need proper mental models to interpret these images in a context. Radiological images can affect the structure of the models the student constructs during learning [38] which influences the performance pattern on how easy or difficult it is to apply the knowledge gained [39]. It is often assumed that including radiological images in the preclinical years of medicine will complicate the topic for the student. Furthermore, this will result in the MCQs being more complicated and discriminative. In the present study, the evaluation with radiological images showed no increase in the difficulty index. The similar difficulty index in both groups shows that the radiological sessions are not an extraneous load to the student. However, this is in contrast with previous studies, which say that the use of an image within a multiple choice question will have a consistent influence on the performance, and this will depend on whether the students considered the images as irrelevant, useful or essential to answer questions [40, 41]. Vorstenbosch et al. 2013 mentioned that the students appeared to have greater difficulty solving cross-sectional illustrations when compared to more straightforward diagrams [10].

Assessment is the DNA of any formal education [42]. One of the essential components of medical education is to measure the acquired knowledge. MCQs are said to form a helpful assessment tool in measuring knowledge recall questions. If the item is carefully constructed, it also can measure thinking skills crucial for a medical graduate [43]. The quality of an individual test item can be assessed by the post-examination analysis [44]. Therefore, it is essential to review the performance and the quality of the items after administering the assessment [45]. The difficulty index is sometimes called the 'easiness index', since a higher number indicates an easier question. The difficulty index and the discrimination index are reciprocally related [46]. In a study conducted by Hunt in 1978, the item difficulty increased since the student had to interpret a visual image [47]. The present study showed that 88% of items in the group with no radiological images and 86% of the items in the group with radiological images showed a discrimination index of 0.2 and above, which clearly explains that there is not much difference in the discrimination index of the items. Therefore, it is a misconception to consider that inclusion of radiological images will necessarily increase the difficulty of exam questions. Some studies also mentioned that Including images in an exam will increase the item difficulty and reduce the speed at which the students process the information, which results in increased testing time and item difficulty [40]. The results we obtained were similar to the findings of a previous study conducted by Phipps and Brackbills in 2009, demonstrating the comparable capability of these two item formats [48].

Reviewing these questions separately and how the student responded to each question provides information on whether the item measures at the correct difficulty level [49]. Table 1 in the result section showed the consistency in the mean difficulty levels in the two groups which were considered in this study. The acceptable *p* values had a normal range with no significant difference, which meant that the *p* values were similar between the two groups. Furthermore, this can explain that adding a radiological image to an item did not increase the difficulty index of the examination, and the students found these questions not too easy and not too challenging. The difficulty level was the same as that of the group, which contained no radiological images.

### Limitations

A few factors limit the results of the presented study. First, it was conducted on only one batch of students. A more extensive study with more image-based MCQs can help substantiate the results on a broader scale. Another limitation is that this study could not look at the long-term impact on the student, which could also be a valuable area for further research.

## Conclusion

This study adds to the growing area of research that supports integrating radiological images into basic sciences. We conclude that introducing radiological images when teaching anatomy must begin from the preclinical years and must be based on the principle of constructive alignment. Recognizing and interpreting an image is essential in an undergraduate medical program. Using these images to test these abilities within high stake examinations ensures authenticity and constructive alignment. This study suggested that the students found it helpful when radiological images were introduced to them when teaching anatomy; compared to the standards suggested in the literature, the mean difficulty and item discrimination values of the two groups were similar. These findings point out that adding radiological images when teaching anatomy does not increase the difficulty of the subject. Finally, the results in this study also allow us to assist the faculty in understanding if any improvement is required when delivering the sessions.

With the introduction of newer teaching strategies and modalities, many changes are taking place in the field of education. These newer modalities will continue to receive attention. Recognition and interpretation of radiological images is an essential skill the student should learn during the preclinical years. Including these images in summative examinations can ensure authenticity and constructive alignment. In the present study, we demonstrated that the addition of radiological images had no overall difference in the various parameters in the item analysis. The study also allows us to improve the integration of radiology into our gross anatomy courses. Furthermore, it can assist the faculty in understanding if there are gaps in the coverage of anatomical concepts.

## Data Availability

The datasets used during the current study are available from the corresponding author on reasonable request.
